# *Acinetobacter calcoaceticus* from a fatal case of pneumonia harboring *bla*_NDM-1_ on a widely distributed plasmid

**DOI:** 10.1186/s12879-015-0870-7

**Published:** 2015-03-18

**Authors:** Peng Li, Chaojie Yang, Jing Xie, Nan Liu, Houzhao Wang, Ling Zhang, Xu Wang, Yong Wang, Shaofu Qiu, Hongbin Song

**Affiliations:** Institute of Disease Control and Prevention, Academy of Military Medical Sciences, 20 DongDa Street, Fengtai District Beijing, 100071 China; 174th Hospital of PLA, Xiamen, Fujian China

**Keywords:** *A. calcoaceticus*, Type IV secretion system, *bla*_NDM-1_, IS*Aba125*

## Abstract

**Background:**

We have recovered one *bla*_NDM-1_-harboring bacterial strain, designated as XM1570, from a sputum sample obtained from a fatal case of pneumonia in China.

**Methods:**

Biochemical profiling, 16S rRNA sequencing and antimicrobial susceptibility testing were performed. Conjugation experiments were conducted to determine transmissibility of resistance. Pulsed-field gel electrophoresis and whole genome sequencing were performed to identify strain-specific features.

**Results:**

The isolate XM1570 was identified as *Acinetobacter calcoaceticus*. Whole genome sequencing identified two plasmids, pXM1 and pXM2. Comparative analysis showed >99% similarity between XM1570 and *A. calcoaceticus* PHEA-2. Plasmid pXM1 carried the carbapenemase gene *bla*_NDM-1_ and displayed high homology with previously described plasmids isolated from different *Acinetobacter* spp., which were collected from human or livestock distributed in China and worldwide. The *bla*_NDM-1_ gene was located on this conjugative plasmid in a transposon-like region flanked by two copies of the insertion sequence IS*Aba125*; and resistance to all tested β-lactams was observed. Transferability of resistance from pXM1 to the transconjugants was identified. Plasmid pXM2 had an insertion sequence IS*Aba125* and a −35 region of the *bla*_NDM-1_ gene promoter but the *bla*_NDM-1_ gene was not present. A chromosomally located carbapenemase-encoding gene *bla*_OXA-75_ was detected; however, this gene was interrupted by an insertion sequence IS*Aba22* belonging to IS3 family.

**Conclusions:**

Location of *bla*_NDM-1_ on different self-transmissible plasmids could facilitate geographically broad dissemination and host range expansion of the *bla*_NDM-1_ gene via horizontal gene transfer. Our findings of this normally environmental species *A. calcoaceticus* XM1570 further underline the significant clinical challenge and the essential need for surveillance including molecular methods and plasmid analyses.

**Electronic supplementary material:**

The online version of this article (doi:10.1186/s12879-015-0870-7) contains supplementary material, which is available to authorized users.

## Background

Carbapenems are recommended antibiotics for the treatment of nearly all *Enterobacteriaceae* in the past but now becoming increasingly ineffective to extended-spectrum β-lactamase (ESBL)-producing multidrug resistant bacterial infections [1,2]. Metallo-β-lactamases (MBLs) that hydrolyze all β-lactams including carbapenems are found with increasing frequency among the *Enterobacteriaceae* and non-*Enterobacteriaceae* from human, animals and the environment [3,4], limiting the effectiveness of antimicrobial therapy. The plasmid-located carbapenem resistance gene *bla*_NDM-1_ was first detected in *Klebsiella pneumoniae* [5]. The gene, often flanked by mobile genetic elements, is now one of the most widespread carbapenemases genes and has been detected worldwide in multiple Gram-negative bacterial species including *Acinetobacter* spp., *Escherichia coli* and *Klebsiella* spp*.* [6]. Globalization and international travel accelerates the rapid dissemination of NDM-1 producers between different countries and continents [7]*.*

Since the first report of a *bla*_NDM-1_ positive *A. baumannii* strain in China, this resistance gene has been observed in different species throughout China [8-13]. In 2011 only four *A. baumannii* isolates out of 11298 clinical Gram-negative bacilli were positive for the *bla*_NDM-1_ gene [9]. More recently studies have reported high isolation rates of *bla*_NDM-1_-containing bacteria from clinical fecal samples and the sewage of hospitals [13,14]. Of concern was the reported identification of 27 *Acinetobacter* spp. isolates with the *bla*_NDM-1_ gene recovered from intensive care units in China [15]. Surveys of the *bla*_NDM-1_ gene in bacteria of food animal origin also recovered two positive strains, *A. lwoffii* and *A. baumannii*, from the cloacal swab sample of a chicken (1 of 146) and the lung sample of a sick pig, respectively [16,17].

The *bla*_NDM-1_ gene was found to be located on different plasmids which are easily transferable and capable of rearrangement [18]. Several *bla*_NDM-1_-harboring plasmids have been reported to be indistinguishable from or highly related to the plasmid pNDM-BJ01 which has been previously isolated from clinical *A. lwoffii* strains [17,19-22]. Analysis of the plasmid pNDM-BJ01 revealed a different genetic context for the *bla*_NDM-1_ gene when compared to other *bla*_NDM-1_ containing plasmids [23]. The *bla*_NDM-1_ gene was found to be flanked by two copies of IS*Aba125* and this common surrounding genetic structure of *bla*_NDM-1_ were shared in most of the non-*baumannii Acinetobacter* spp*.* across China [24]. Although it is not clear how the *bla*_NDM-1_ gene has emerged and spread across China, the diversity of *bla*_NDM-1_ harboring species recovered from different locations and the repeated occurrence of pNDM-BJ01-like plasmids are unlikely to be a coincidence [19].

To our knowledge, no NDM-1-harboring *A. calcoaceticus* has ever been isolated and identified to be related to a serious human infection. Here we describe the detection and genetic characterization of a *bla*_NDM-1_ harboring *A. calcoaceticus* strain XM1570.

## Methods

### Case report and bacterial isolates

During routine sentinel surveillance, we isolated a *bla*_NDM-1_-harboring bacterial strain from a patient died of pneumonia and respiratory failure. The isolate was positive for the *bla*_NDM-1_ gene and identified as *A. calcoaceticus,* which is a rare human pathogen but a species frequently recovered from soil and water with no implication in serious human diseases [25]. *A. calcoaceticus* XM1570 was isolated from the patient described above. Other 22 *Acinetobacter* spp. isolates were recovered from sputum samples of patients in the same hospital during May-July 2010. All isolates were identified by a combination of API identification system (BioMerieux, Marcy l’Etoile, France) and 16S rRNA sequencing using previously described primers [26]. PCR was used to screen the *bla*_NDM-1_ gene and the *bla*_OXA-75_-like genes [27,28]. Samples were collected as part of standard patient care. Informed consent was not obtained except included patients were subjected to extra procedures. Collection of all samples in this study was approved by the ethics committee of the Academy of Military Medical Sciences (China).

### PFGE analysis

Genetic relatedness of the isolates was analyzed by *Apa*I-macrorestriction genomic DNA and pulsed-field gel electrophoresis (PFGE); pattern analysis was determined using BioNumerics software version 6.0 (Applied-Maths, Sint-Martins-Latem, Belgium) as previously described [14]. The Dice coefficient of similarity was calculated with a position tolerance of 1.2% and an optimization of 0.3%, and the dendrogram was constructed by using the unweighted-pair group method using average linkages (UPGMA).

### Susceptibility testing and conjugation experiment

Strain susceptibilities to different antibiotics of XM1570 were assessed by the Microscan WalkAway 96 SI identification system (Dade Behring, Newark, USA) in the hospital. Antimicrobial susceptibility testing of all isolates was determined by using disk diffusion method. The minimal inhibitory concentrations (MICs) of the recipients and the transconjugants were then determined by using the Sensititre™ semi-automated antimicrobial susceptibility system (TREK Diagnostics, Inc., Westlake, OH, USA) and the Sensititre™ Gram-negative custom plate PRCM2F according to the manufacturer’s directions. The E-test strips were used as necessary to determine the MICs of certain antibiotics such as imipenem. The above results were interpreted according to the Clinical and Laboratory Standards Institute guidelines [29]. Additionally, The MBL Etest strips (AB bioMerieux, Sweden) were applied to confirm the MBL production.

Conjugative experiments were performed using *E. coli* J53 Azi^r^ as a recipient [5]. The protocol was modified as follows: Overnight cultures of the donor strain (20 μl) and recipient strain (60 μl) were mixed with 2 mL of fresh Luria-Bertani broth and incubated for 4 h at 37°C. The mixture was plated on MHA plates containing ampicillin (100 mg/L) plus sodium azide (250 mg/L) for counter selection for 24 h at 37°C. Bacterial colonies were transferred to broths and incubated for 7 h at 37°C. DNA templates were extracted with TIANGEN Bacterial Genome Extraction Kit (TIANGEN, Beijing, China). Transconjugants, selected for by growth on ampicillin, were confirmed by 16S rRNA sequencing and transferability of the *bla*_NDM-1_ gene was verified by PCR and sequencing.

### Genome comparison and phylogenetic analysis

The whole genome of *A. calcoaceticus* XM1570 was sequenced and assembled as described previously [30]. Protein sequences of 18 *Acinetobacter* spp*.* strains with complete genomes were downloaded from the NCBI FTP (ftp://ftp.ncbi.nih.gov/genomes/Bacteria/) and compared with those of *A. calcoaceticus* XM1570 to generate core conserved proteins, which were defined with a minimum of 95% identity and coverage. Those conserved proteins were then concatenated and aligned by ProbCons with default options [31]. PhyML 3.0 [32] was used to construct the phylogenetic tree using the Maximum-Likelihood method. The tree was bootstrapped 1000 times to estimate the confidence of tree topologies. The graphic representation was performed and manually edited with FigTree (http://tree.bio.ed.ac.uk/software/figtree/). To further assess genome structure and rearrangement, sequences of XM1570 were compared with the only finished genome of *A. calcoaceticus* strain - *A. calcoaceticus* PHEA-2, and aligned sequences were visualized using the Mauve software (version 2.3.1) [33]. Putative orthologs between XM1570 and PHEA-2 are defined as proteins having a minimum of 50% identity and 50% coverage of the query with a maximum E-value threshold of 1 × 10^−5^ and detected by reciprocal best blast hits. Genome sequences of plasmids pXM1 and pXM2 were compared with sequences of the NCBI database (BLAST search) respectively. Sequences of plasmids with high homology were downloaded from NCBI, such as p3ABAYE [GenBank: CU459140], pMS32-1 [GenBank: KJ616405], pNDM-BJ01 [GenBank: JQ001791], pNDM-BJ02 [GenBank: JQ060896], and pNDM-AB [GenBank: KC503911].

## Results

### Microbiological and genetic characterization of strain XM1570

All isolates were identified as *Acinetobacter* spp*.* by using the API system. Isolate 10051442 and 10051570 were further identified as *A. calcoaceticus* by 16S rRNA sequencing while others are identified as *A. baumannii*. Among the 23 isolates, only isolate 10051570 (designated as XM1570) was found to be positive for both the *bla*_NDM-1_ gene and *bla*_OXA-75_ gene.

**Figure 1:**
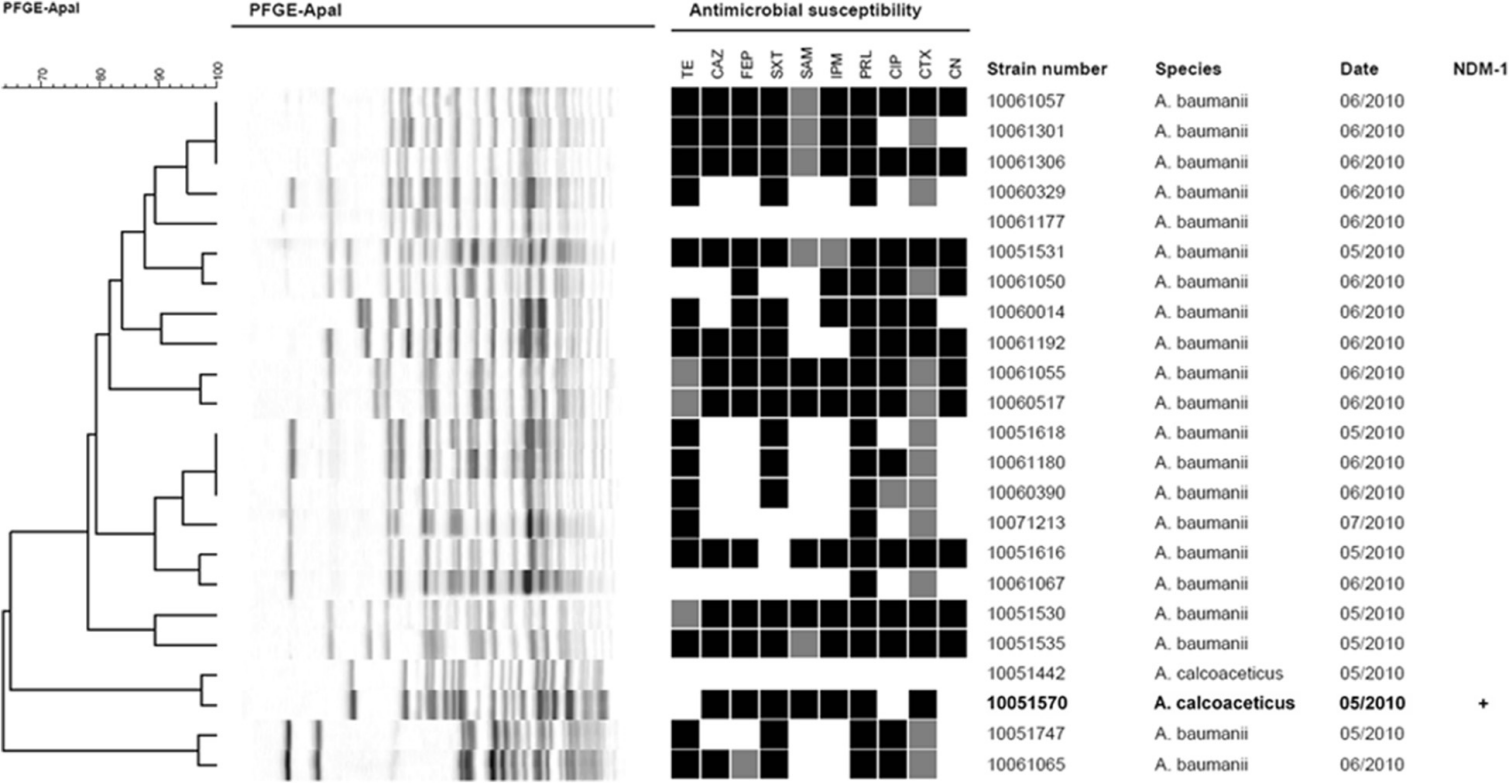
**PFGE-based dendrogram and antimicrobial resistance profile of the 23 isolates from the same hospital in Xiamen, Fujian, China.** The dendrogram was constructed based on unweighted pair-group method using average linkages and pairwise Dice coefficients. Antimicrobial susceptibility was performed by disk fusion, black indicates resistance; gray indicates intermediate; white indicates susceptible. Antibiotics are tetracycline (TE), ceftazidime (CAZ), cefepime (FEP), trimethoprim/sulfamethoxazole (SXT), aztreonam (SAM), imipenem (IPM), piperacillin (PRL), ciprofloxacin (CIP), cefotaxime (CTX) and gentamicin (CN). The strain number, isolate date, species and NDM-1 phenotype of the strains are shown.

PFGE was performed to investigate the population structure of the *Acinetobacter* isolates. All isolates clustered into three groups at an 80% similarity level. Isolates XM1570 and 10051442 clustered separately from the other isolates, and shared 97% PFGE patterns with difference of two electrophoresis strips (Figure 1). However, the profile of antimicrobial susceptibility was quite different between these two isolate. Isolate 10051442 was susceptible to all of the 10 tested antibiotics, while XM1570 demonstrated resistance to all tested β-lactams including carbapenems, but still susceptible to amikacin, genatamicin, minocycline, tigecyline and colistin (Table 1, Figure 1). Resistance to imipenim was further confirmed by Etest, and MBL production was also observed in XM1570. Most of other isolates also displayed multidrug resistances, eleven of which also showed resistance or intermediate resistance to imipenem.

**Table 1:** **Antimicrobial susceptibility profile of** ***A. calcoaceticus*** **XM1570**

**Antimicrobial agents**	**Susceptibility** ^**a**^	**MICs (mg/L)**
Ampicillin	R	>32
Piperacillin	R	>128
Amoxicillin/clavulanic acid	R	>32/16
Ampicillin/sulbactam	R	>32/16
Piperacillin/tazobactam	R	>128/4
Cefotaxime	R	**≥**64
Cefoxitin	R	>32
Ceftazidime	R	>32
Cefepime	R	>32
Cefazolin	R	>32
Cefoperazone	R	>64
Ceftriaxone	R	>64
Cefuroxime	R	>32
Imipenem	R	>16
Meropenem	R	>16
Amikacin	S	**≤**16
Gentamicin	S	≤1
Ciprofloxacin	R	>4
Levofloxacin	R	>8
Tetracycline	I	8
Minocycline	S	≤4
Tigecycline	S	≤1
Aztreonam	R	>32
Colistin	S	≤2
Trimethoprim/sulfamethoxazole	R	>4/76

^a^S, susceptible; I, intermediately resistant; R, resistant.

The *bla*_NDM-1_-harboring plasmid pXM1 was transferable to *E. coli* J53, which was demonstrated through PCR amplification of the *bla*_NDM-1_ gene. The transfer frequency of pXM1 to *E. coli* J53 ranged from 9.4 × 10^−3^ to 1.14 × 10^−2^ per donor cell, a relatively high transfer frequency like pNDM-BJ01 which has been previously noted [20]. Transconjugants carrying the *bla*_NDM-1_ plasmid were resistant to ceftazidime, cefazolin, cefoxitin, ticarcillin, ampicillin and ticarcillin/clavulanic acid, but still susceptible to imipenem, furadantin, cefepime, levofloxacin, aztreoname and trimethoprim/sulfamethoxazole (Additional file 1: Table S1). However, the level of resistance to imipenem in the transconjugants was increased from 0.3 mg/L to 1.4 mg/L according to the Etest results.

### Comparative and phylogenetic analysis of *A. calcoaceticus* XM1570

**Figure 2:**
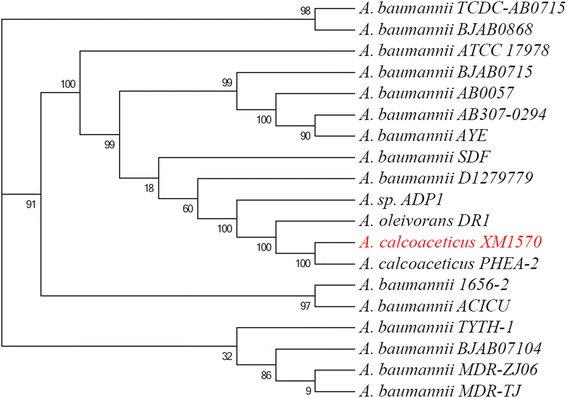
**Phylogenetic tree of** ***A. calcoaceticus*** **XM1570 and other** ***Acinetobacter*** **spp..** Bootstrap values are indicated at the nodes as percentages of 1000 replications.

By comparing proteins of the 18 finished *Acinetobacter* spp. genomes with those of *A. calcoaceticus* XM1570 [GenBank: AMXH00000000], a whole genome phylogeny was constructed based on concatenated sequences for 220 conserved proteins. On the basis of sequence similarity, XM1570 was clustered with non-NDM-1 producing *A. calcoaceticus* PHEA-2 (Figure 2), an isolate recovered from industry wastewater in China [34]. The high bootstrap values (100%) confirmed the cluster of the two sequences was robust. This observation is consistent with a recent study that found soil isolates, including *A. calcoaceticus* and *Acinetobacter* sp. DR1, grouped into one environmental clade and are not involved in serious human infection [35].

**Figure 3:**
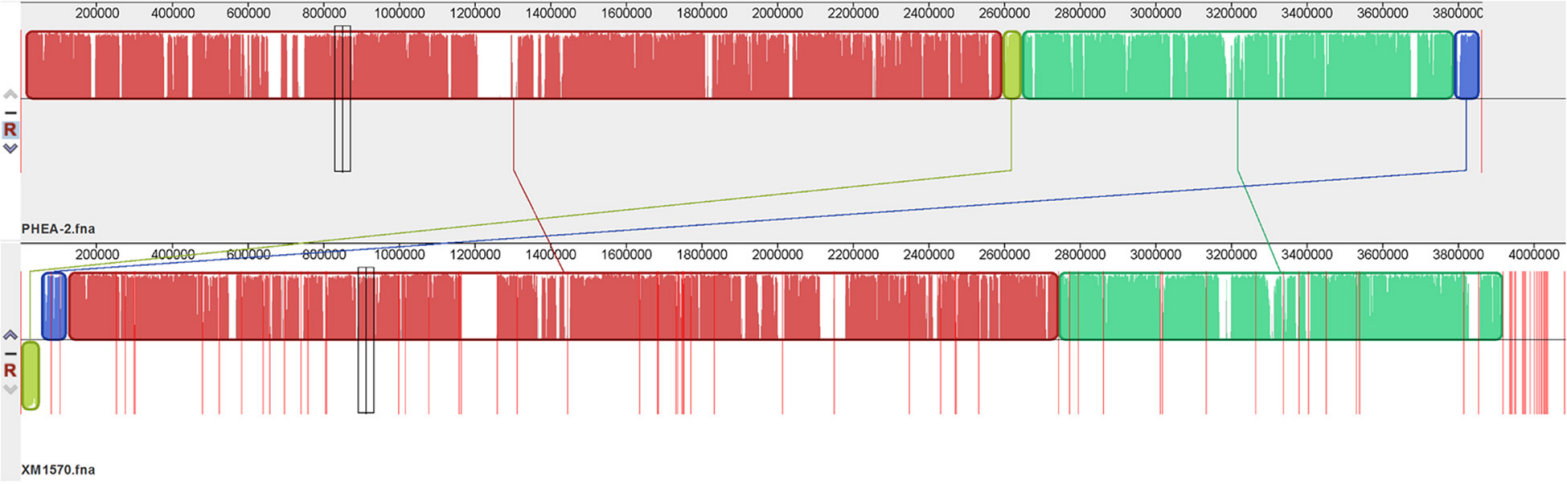
**Mauve alignment of** ***A. calcoaceticus*** **PHEA-2 (top) and XM1570 (bottom) genomes.** Regions that are homologous in each genome are represented by same colors and connected by lines.

The genome of XM1570 was aligned and compared to that of PHEA-2, and showed high level of synteny between these two sequences (Figure 3). Comparative analysis revealed XM1570 and PHEA-2 shared a set of 3148 orthologous proteins corresponding to 83.3% (3148/3781) and 87.5% (3148/3599) of all proteins, respectively. The intrinsic *bla*_OXA-75_ gene of strain PHEA-2 was also found in XM1570 but was disrupted by an IS*Aba22* insertion sequence belonging to the IS3 family.

### Characterization of *bla*_NDM-1_-harboring plasmid pXM1

**Figure 4:**
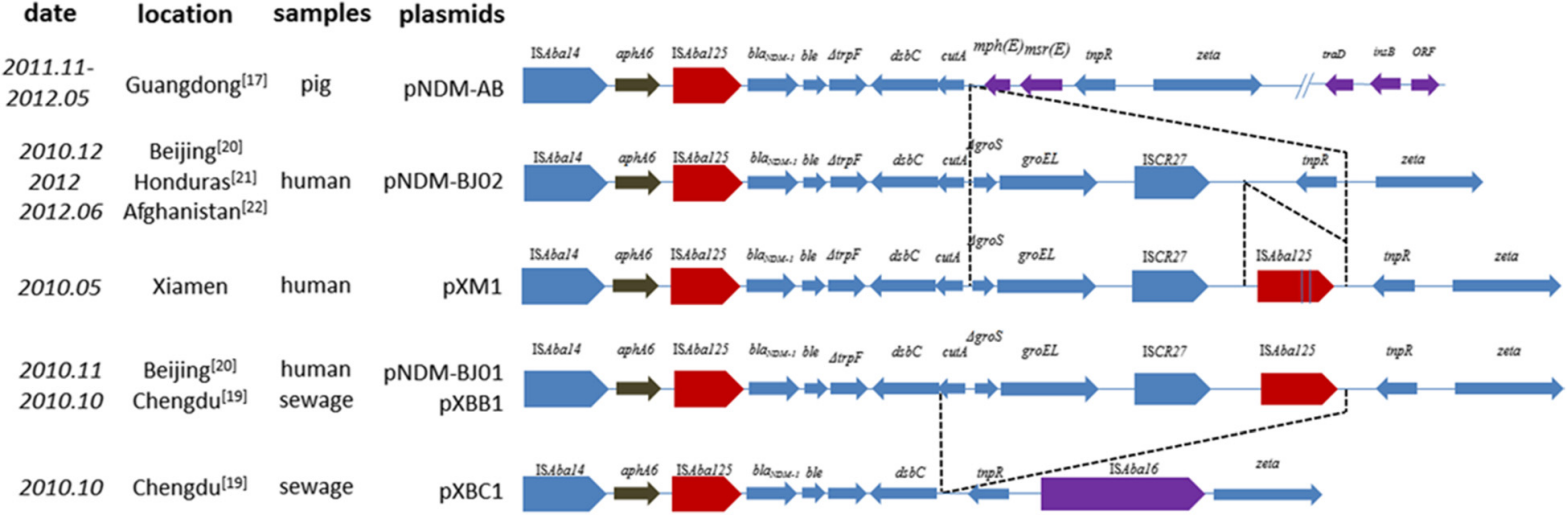
**Schematic representation of** ***bla***_NDM-1_ **-carrying plasmids isolated from different locations. ORF, open reading frame.** The dashed line indicates the loss of genes in the corresponding location of the plasmids. Purple colored blocks represent the gain of genes. Arrows indicate gene orientation from 5′ to 3′. Length of the arrow represents relative gene sizes.

The complete sequence of the *bla*_NDM-1_-carrying plasmid pXM1 [GenBank: CM001802] has 47,274 bp in length with an average G + C content of 40.8% and contains 55 predicted coding sequences. A BLAST search showed that pXM1 shared >99% identity to plasmid pNDM-BJ01 recovered from a clinical isolate of *A. lwoffii* in Beijing (Figure 4) [20]. There are only two nucleotide differences between these two plasmids, located at 17688 and 17760 in the region of the second insertion sequence IS*Aba125*, adjacent to transposase gene IS*CR27*.

The *bla*_NDM-1_ gene in plasmid pXM1 was flanked by two copies of IS*Aba125* inside a composite transposon *Tn125* as previously described [36]. *Tn125* often co-exists with the *aphA6* gene and locates downstream on the plasmid [22,26]. Susceptibility testing showed that both XM1570 and the J53 transformants were susceptible to amikacin as previously observed in *A. lwoffii*, which was suggested to be a result of disruption in the promoter sequence of *aphA*6 [20,24]. Similar to plasmid pNDM-BJ01, pXM1 also contains a region of approximately 14 kb containing genes for a type IV secretion system (T4SS).

A number of studies have reported the occurrence of a plasmid similar to pXM1 including pXBB1 and pXBC1 isolated from hospital sewage in Chengdu (2010) [19], pNDM-BJ01 from patients in Beijing (2010) [20], pNDM-AB from pigs in Guangdong (2011 and 2012) [17] and pNDM-BJ02 from patients in Beijing (2010) [20], Honduras (2012) [21] and Afghanistan (2012) [22]. These plasmids share similar sequences with a few gene additions and subtractions associated with IS*Aba125* downstream of *bla*_NDM-1_ (Figure 4). In addition, pNDM-AB also obtained a short gene segment located downstream of the P-type T4SS. It is noteworthy that these *bla*_NDM-1_ containing plasmids appear to have emerged at a similar time period although at different geographic locations and host origins with no obvious epidemiological link. The repeated occurrences of pNDM-BJ01-like plasmids suggest a potential mechanism for fast dissemination of the *bla*_NDM-1_ gene through the community as well as in livestock and in the environment. More investigations are necessary to determine whether the plasmid is distributed among other bacterial species in China; greater attention should be paid to the surveillance of the *bla*_NDM-1_-carrying plasmids in the future.

### General features of plasmid pXM2

pXM2 [GenBank: CM001803] was assembled with an unknown gap and has a length of 93,891 bp with an average GC content of 37.7% and 89 predicted protein coding sequences. A BLAST search indicated that pXM2 had 99% identity with plasmid pMS32-1 recovered from *A.* pittii (unpublished data) in Taiwan and p3ABAYE recovered from *A. baumannii* AYE in France [37]. pMS32-1 and p3ABAYE shared >99% identity and 100% coverage. All three plasmids contains an IS*17*-like element (94% amino acid identity) which was found downstream of the *bla*_RTG-4_ gene on a new transposon *Tn2014* [38,39]. Genome comparisons revealed that p3ABAYE contains an incomplete sequence of the transposase *insB* and an approximately 4 kb gene encoding a putative phage-like protein; these were not present in pXM2. This plasmid acquired two copies of the transposase of IS*Aba22* (nucleotides 55158–56426, 65400–66643) with an 837 bp common region and a copy of the transposase of IS*1236* (nucleotides 57615–58848) respectively, both belonging to the IS3 family. However, pXM2 has also gained a region containing a simple insertion of IS*Aba125* (1087 bp), which provided the −35 region used by *bla*_NDM-1_, flanked by 3 bp DR.

## Discussions

Frequent international air travel and multiple healthcare facilities further contribute to the rapid dissemination of the *bla*_NDM-1_ gene, which should become the focus of global concern for treatment and public health [7]. Infections caused by *Acinetobacter* spp*.* are difficult to treat because of their intrinsic multidrug-resistance and readily acquired new resistance mechanisms. A recent study has also suggested that most nonself-transferable plasmids of Acinetobacter could be transmitted among strains of *A. baumannii* through the *rep*Aci6 gene [40]. Moreover, these closely related *bla*_NDM-1_-harboring plasmids, such as pXM1 and pNDM-BJ01, displayed a different genetic context of the *bla*_NDM-1_ gene from others isolated outside of China. Epidemiological investigation showed that infected patients did not report a history of travelling internationally or even domestic travel. Though the pXM1-like plasmids has a high transferability, it remains unclear whether the isolate in our study acquired such plasmid from human transmission or animal food by horizontal gene transfer. In this study we found that isolates 10051442 and XM1570, collected from the same hospital, showed the similar PFGE patterns, indicating that they may belong to a closely related clone. However, there was considerable discrepancy between antimicrobial susceptibility results. The reason for the difference observed needs further elucidation.

The situation with respect to overuse of antibiotics and antibiotic resistance in China is severe. The mean prevalence of resistance among hospital-acquired infections is as high as 41%, and among community-acquired infections it is 26%, and China also has the world’s most rapid growth rate of drug resistance [41]. However, the plasmid pNDM-BJ01 was found to be unstable and is readily lost if antibiotic pressure is reduced, which implied a limited expansion potential in wild *A. lwoffii* strain [42]. A large scale survey also observed that the plasmids of *bla*_NDM-1_ positive transconjugants are prone to be lost in the absence of imipenem selection [13]. More attention to the misuse or overuse of antibiotics to prevent the epidemic spread of *bla*_NDM-1_-associated organisms in China is critical to control. A valid pharmaceutical policy that includes a strategy for rational drug use and effective control of nosocomial infection should be formulated. Based on phenotypic properties alone, members of *A. calcoaceticus-A. baumannii* complex (ABC) are difficult to distinguish from each other. *A. calcoaceticus* is excluded when addressing epidemiological issues related to *Acinetobacter* [43,44]. Although the *bla*_NDM-1_ gene was mostly identified in prevalent species of *Enterobacteriaceae*, this study suggests that we should also strengthen the surveillance for uncommon non-enteric opportunistic strains.

Plasmids harboring the P-type T4SS are often broad host-range [45] and pXM1 also contains a region for conjugative transfer and plasmid replication. The plasmid-located *bla*_NDM-1_ gene could readily transfers across species boundaries. So it is critical to investigate whether this frequently emerging plasmid can transfer to virulent pathogens, which could pose a great threat to humans. Systemic surveillance should be extensively carried out for monitoring this kind of multidrug resistance bacteria in China.

## Conclusion

In this paper we outlined the detection of a *bla*_NDM-1_ harboring *A. calcoaceticus* strain. This isolate, like most of the reported *Enterobacteriaceae* isolates with NDM-1, was multidrug-resistant to many antibiotic classes. Plasmids like pXM1 have been recovered from different geographical regions worldwide during same time period. Our findings suggest that the emergence of this rare environmental strain once again raises the threat of the potential transfer of *bla*_NDM-1_ carbapenemase to other species, potentially creating an opportunistic pathogen.

## Additional file

Additional file 1: Table S1. Antimicrobial susceptibility profiles of *A. calcoaceticus* XM1570 and the transconjugants *E. coli* J53.
