# Supplementary material for: Acinetobacter calcoaceticus from a fatal case of pneumonia harboring blaNDM-1 on a widely distributed plasmid
Source: BMC Infect Dis. 2015 Mar 18;15:131. doi: 10.1186/s12879-015-0870-7 (PMC4373515; doi:10.1186/s12879-015-0870-7)
Supplement: Additional file 1: Table S1. — Antimicrobial susceptibility profiles of A. calcoaceticus XM1570 and the transconjugants E. coli J53. [file 12879_2015_870_MOESM1_ESM.doc]

**Supplementary table S1. Antimicrobial susceptibility profiles of *A. calcoaceticus* XM1570 and the transconjugants *E. coli* J53.**

| **Antibiotic** | **Susceptibility** | | |
| --- | --- | --- | --- |
|  | **XM1570** | **transconjugant *E. coli* J53** | ***E. coli* J53** |
| Ceftazidime | R | R | S |
| Ceftriaxone | R | I | S |
| Imipenem | R | S | S |
| Furadantin | R | S | S |
| Piperacillin | R | I | S |
| Tetracycline | I | S | S |
| Cefepime | R | S | S |
| Cefoperazone | R | I | S |
| Cefazolin | R | R | S |
| Cefoxitin | R | R | S |
| Tobramycin | S | S | S |
| Levofloxacin | R | S | S |
| Gentamicin | S | S | S |
| Ticarcillin | R | R | S |
| Aztreonam | R | S | S |
| Ampicillin | R | R | S |
| Chloramphenicol | I | S | S |
| Trimethoprim/sulfamethoxazole | R | S | S |
| Norfloxacin | S | S | S |
| Amikacin | S | S | S |
| Ticarcillin/clavulanic acid | R | R | S |

**^a^ S, susceptible; I, intermediately resistant; R, resistant.**
